# Supplementary material for: Patterns of Intron Gain and Loss in Fungi
Source: PLoS Biol. 2004 Nov 30;2(12):e422. doi: 10.1371/journal.pbio.0020422 (PMC532390; doi:10.1371/journal.pbio.0020422)
Supplement: Table S1 — Also available at http://genes.mit.edu/NielsenEtAl/. (4.3 MB ZIP). [file pbio.0020422.st001.zip › NielsenEtAl/html/1349.html]

AN4435.1.NCU03360.1.MG03232.1.FG00627.1


```
 CLUSTAL W (1.82) Multiple Sequence Alignments - Introns Inserted


Sequence 1: MG03232.1	1040 aa
Sequence 2: FG00627.1	979 aa
Sequence 3: NCU03360.1	951 aa
Sequence 4: AN4435.1	980 aa
Alignment Length: 1113 aa
Number Identitical Residues: 241 aa
Alignment Score (without introns) 13940


MG03232.1 	MDRTGDRPALSLSNG-----GRGLPENPAQGRRNMTNGGANGGGSLNSLPPMPTAIPANV
NCU03360.1	----------------------------------MPPVGPHGNH-----LPHPP------
FG00627.1 	MERPDRLAGSSVGSTSQASIPRSLPENHTSGRRPVPNSAATSNG-----PPPPPPIPS-L
AN4435.1  	MISTLVRPEDSLRMPRSYRDTEEEDANRHSPEPAAPLSSSSSTTDGSNKSEDEATLVN--
          	   .   .  :     .         .  .     .  .. .  . ..     ..     

MG03232.1 	RSMSSTSSSTLSANPPVSAGQVIGMARRAMAEALKEHESQAAEASGVSNELKPGVTIDLS
NCU03360.1	--HRDASNSSLSASPPLSSNQVIALVRESMRVAHEN-EAKATEG---ISTLKPGLTIDLS
FG00627.1 	RSVPSSSSLSTVG---MSAAQVINLAREAMQNAIES-ESQAAEAGAVSTGLRTGVTIDLS
AN4435.1  	GSTGDTSSRPTKSRESLTPEETIQLARRAVENGIQETKRSLAGSEAVGDVVKPKLTIDLG
          	 :  .:*. .  .  .::. :.* :.*.::  . :. : . : . .    ::. :****.

MG03232.1 	RKNIQELPEEIVDVIKHELER2---------------------LALSHNKISSFPARWSE
NCU03360.1	RKRIQKLPDEVVDIIKDELER2---------------------LALSHNYIQTFPARFSE
FG00627.1 	RKGIQKLPDEVVDIVKGELER~SASKNSSSNTRPDADTYTVSRLALSHNQLTSLPARFSE
AN4435.1  	HSNISRIPEPVVDIIKDEVER2---------------------LSLWNNQLVHIPYRFAE
          	:. *..:*: :**::* *:**                      *:* :* :  :* *::*

MG03232.1 	CSSLRYLNVRNNELREFPLP0LCHLKSLEILDLGRNKIRSLPPEIVKLTSLKVFAVQKNR
NCU03360.1	CTSLRYLNVRSNRIKEFPLA0LCDLKSLEILDLGRNMLRVLPPDIVKLSSLKVFSIQKNE
FG00627.1 	CTSLRYLNIRGNQIKEFPMP0LCELKSLEILDLGRNSLRILPADIARLSSLKVLSIPKNQ
AN4435.1  	CSHLRYLNVRSNNFREFPRG0VIKLPLLEILDLSRNKISQLPEEIKKLTSLRVLSVMQNR
          	*: *****:*.*.::***   : .*  ******.** :  ** :* :*:**:*::: :*.

MG03232.1 	IEELPICLADMGSLQMIKLEGNPIRFPPPEVLQVQAASPPNEGHLKESEVTELTVTAHIK
NCU03360.1	VSELPLCLADMPSLSVIKLEGNPLVFPPPQVFQVPPPSVQNEGILKESELTEITVTAHVK
FG00627.1 	IRELPLCIADMGSLQVIKFEGNPISFPPRDAIQVQAASPPHEGIQKDNEVTEVAVTLQIK
AN4435.1  	LDDLPLGVSDMNKLQILKVAGNPLRYPLRKVIETSEAEITSS--MMSDNEKEVALTAELK
          	: :**: ::** .*.::*. ***: :*  ..::.  ..   .    ..: .*:::* .:*

MG03232.1 	KFMKQRALELTSSRSDSEMGMDESN~EGMETPRVPIKRVVSGRFPVRVNGAGLPDLRSPA
NCU03360.1	KYLKATANS-ILEREQAESPNDE-T~DGIETPRPTIKRVFSGRFPIKVNGTDVPDLRSPN
FG00627.1 	RYLKQYTIN---GRSESDNTGDESS~EGAETPRFPLKRVASGRFPVKVQGADLPDIRSPN
AN4435.1  	RYLKARQPI-----NSNDFESNNES2DGILDTPKPVKRGVSSRFPVIPSTGDGADPKSPS
          	:::*          .. :   ::.. :*   .  .:**  *.***:  .  . .* :** 

MG03232.1 	FARPGPPPIPTRSHYRGLSQQNTAPRKPGVMPLTIGNVNERMRSNSETL-SQTPRADRPG
NCU03360.1	LI--RPPPIPSRSHYRGLSQQSSAQRRPGVMPLTIGNPNERIRSNSETTGAHHSSRERSE
FG00627.1 	IG-SRAPPIPSRSHYRGLSQQNTAFRRPGVMPLTIGNVNERVRSNSESL-LRAERSDG--
AN4435.1  	LS--RPPPIPLKSHYR-IASGHGGALQILQRPGPIPGANERNRSNSEGI-----IQASFA
          	:    .**** :**** ::.   .  :    * .* . *** *****             

MG03232.1 	ERSRRMGVVSQKAQQLGTLDETQANNRFS-HYRGLSHGSAMQGNGQMLNGK--MPVSPAE
NCU03360.1	SRSRRMGIVSRKGSELGTLDELEAKDRFSIHYRGLSHGSAMQPGNGSVPGQSTAPSSPAD
FG00627.1 	-RSRRMGIVSRKPSDLGTLDEMQVNNRFS-HYRGLSHGSAMQGN--QATTK--SPATPTE
AN4435.1  	TRSKRMGVISRKNTDLGTLDEMRP-YRNS-HLRGLSYGSILRTRPSISNSS--SPSSPRE
          	 **:***::*:*  :****** .   * * * ****:** ::        .   * :* :

MG03232.1 	SI-LQRPIYVRRLSILPERRRESKFVDPVLEAAKGVLYSIFQIHPMIQMLMGLTSDGLAK
NCU03360.1	SISLQRPIYVRRLSILPERRRESKFIDPVLEAAKGILYSIFQIHPMIQMLMGLANDGSSK
FG00627.1 	PY-LARPIYVRRLSILPERRRESKIFDPVIETAKGILYSVFQIHPMIQALMGLTSDGSAK
AN4435.1  	RR-RPRDGFVNRMSSLPEHKGERETESPIIESAKGILFALFQVQSHVYALINVIKRDDYR
          	     *  :*.*:* ***:: * :  .*::*:***:*:::**::. :  *:.: . .  :

MG03232.1 	RSSLEIVFYNTNSHVEELEQEIQRHEADEDG---ASQFENENVMRACITLVNAYVHVCSL
NCU03360.1	RSSLEIVFYNTNAHVEQLELEIQKHDQAGDG---GGPRENENVQRACITLVQAYSHVCSL
FG00627.1 	RSSLEIVFYNTNSHVEELEQELQKHENALGEPLDHSQRENENVHRACQTLVSAYGHVCTL
AN4435.1  	RNSLEIVFYNASTHVDRLNEALENAENSRADDAEPMRASYEAVKRECETCIMAYSHVGTQ
          	*.********:.:**:.*:  ::. :       .    . * * * * * : ** ** : 

MG03232.1 	LASNVDLFIDNGDPRYIRTLMMQLYNSITELRVTTSHVLPEEGHR-RAASRTAMN----S
NCU03360.1	LANNVDLFLDNGDPRYIRTLLTQLYNSIMELRVTCSQPSPGMPSR-NVPPVPPVPHRLEL
FG00627.1 	LADNIGTFVDFGDARYIRSLLMSLYNSIMELRVTLSSVSAADVRR-RGGPKGGSTG---E
AN4435.1  	LRNSLDRIVANGDSRYIRSLMLMIFGSVVELRNACACLEVPVGNRPRPTGRPPVPEISRE
          	* ..:. ::  **.****:*:  ::.*: *** : :        *..             

MG03232.1 	GTIRPRSRENTATPTAERPN--GIPRSVARGNLTHNPSNLR--------VTTDVPLS---
NCU03360.1	ETIRPHSRDNSVTPTIDRPP--QTAR-LRNPGPMHPHANLR--------VATDVPLM---
FG00627.1 	RTARPHAREPSATPTLSRSN--GARN--RNGTLVHHPVNLR--------VSTDVPVPTPL
AN4435.1  	SADSDRYHCTTVTPTRGREPSFSTRRLRSDTTILHPQTNMHGPLPASATFQSAVSSPGFA
          	 :   : :  :.***  *  :    .        *   *::.. .::::. : *.     

MG03232.1 	SIPYVNGTGRTATISS-----------ATPRSGESFVSTGSRGLSGD-------FTEEDR
NCU03360.1	--PYNNGMHR-QVMSA-----------VTPRSGDPFP-MSAGGMRMP-------SDEEDR
FG00627.1 	SATYLNGNGRTATLAS-----------ATPRSGESFSSIGSRGMSSE-------FTEEDR
AN4435.1  	PTPYSYGARSRSSSRSNHVNTSVPSSLATPRSGESFPPMPTSVIPKINPLTGLDEIEEER
          	. .*  *        :.  .:: .:: .*****:.* .  :  :   .. :. .  **:*

MG03232.1 	IFENIFLALQKSTDLVVRLLPAFNHQYTNGLQAAIQHRN-TRESQMWKSLISKCSMTIQQ
NCU03360.1	IFERIFLALMKSADLALKVLPNLSAQFSVALRNATTQRASDHVVHCWKALIVKCNDSIRH
FG00627.1 	QFERIFLALQRSTDLVMGTLPNFNAQLAGGLRNAMYQRTPESVLQHWKQLIAMCGQVIQQ
AN4435.1  	IFEKIFYQLTAAYTAALQALPVARRHFARNLEVAEQNREFEDIQMLWNNLIHRCRVCLEV
          	 **.**  *  :   .:  **    : :  *. *  :*        *: **  *   :. 

MG03232.1 	TEKLKTRLSLIKLKEPG--IRTQGSFWNDVRDFVDSWAEFGSKVREVEKPLSLPADTRMR
NCU03360.1	TEILRERLSTIKLKEPG--IRGQPGFWRMCNDYIDAWYVLGKKTMQFQTDIQLPVDAKSR
FG00627.1 	TEILKNRLSMIKLKEPG--VRTQANFWSLCSNFSHSWADMISQIKYGPNSVPLPPDTRAR
AN4435.1  	SEALGLRLSNMKVKEPGGGMRNQREFWQLCKAFMQSFVELVTDMREVRSMHLLPSEVIVI
          	:* *  *** :*:****..:* *  **     : .::  : ..     .   ** :.   

MG03232.1 	LRPFQKSIQEASSLILRSPWGYWLRPGLATSHQTGNGGGIFSPGGAVESHNSTGLNGDRQ
NCU03360.1	MRPIQKAMKDAWELMVHSPWSHFLKPQPSSGGHGRGRMRGRMPGPGMEGVEDTS------
FG00627.1 	LRPIQQSIKETSNIIYKSPWHYLFHPSGAMNGQAYPRG--MSP-----------------
AN4435.1  	LRPVQKASREAGRLIEASPWSYLADMAPGNGPPAIYGPPLPSQTSHQQQQHHHH------
          	:**.*:: :::  ::  *** :      . .                .            

MG03232.1 	YQQNGQYGNPSHNHLHHNPHIQLPMTPQSAALGPAAQATVPATPQSGSFAAALTGNVFER
NCU03360.1	---------SPYSTHGTNTPVLMPMTPQSAALGPAVQATVPSTPKNSSFSAAFSGGLFER
FG00627.1 	--------------------TSVPITPQSAALGPAMQATVPTTPSNSSFAAAFHGNVFDR
AN4435.1  	-----HQQHHPQLNTSMSPSVALPATPLSAALGPAAQATIPSTPASAYSDKFFEGDVFQR
          	          .      ..   :* ** ******* ***:*:** ..     : *.:*:*

MG03232.1 	ADALMSQGGPSSIHSHSRHGTFTSSTSGSSGGMSGSFNSISSTLSSATSYNDDRMGGHSA
NCU03360.1	AETLIQMGG-----------------PGMG-SRSGTMQS--------SSAASLNSIGTTL
FG00627.1 	ADALMAN-------------------PGISMGRNGAFG-------------KGHMGFSSL
AN4435.1  	ADSLLSMPN---------------------------------------------------
          	*::*:   .                                                   

MG03232.1 	SSSSSVISPGGLGGGPGPMASAFPFRMQNGGGRVAL
NCU03360.1	SGDSAMTPNSALSPGPGSLT-PLPFRMN--SGKVGL
FG00627.1 	SSLSSMSSDEGILPTHGPLG-PVPLRLN--GGKVAF
AN4435.1  	--QAPFFSRR--------------------------
          	   :.. .
```
